# A New Mental Health Mobile App for Well-Being and Stress Reduction in Working Women: Randomized Controlled Trial

**DOI:** 10.2196/14269

**Published:** 2019-11-07

**Authors:** Cássia Canha Coelhoso, Patricia Renovato Tobo, Shirley Silva Lacerda, Alex Heitor Lima, Carla Regina Camara Barrichello, Edson Amaro Jr, Elisa Harumi Kozasa

**Affiliations:** 1 Hospital Israelita Albert Einstein Sao Paulo Brazil; 2 Natura Cosméticos SA Cajamar Brazil

**Keywords:** stress, psychological, mental health, health promotion, mobile applications, mind-body therapies, meditation, behavioral symptoms, behavioral medicine, psychology, women’s health

## Abstract

**Background:**

Although the availability and use of mobile mental health apps has grown exponentially in recent years, little data are available regarding their efficacy.

**Objective:**

This study aimed to evaluate the effectiveness of an app developed to promote stress management and well-being among working women compared with a control app.

**Methods:**

Female employees at a private hospital were invited to participate in the study via mailing lists and intranet ads. A total of 653 individuals self-enrolled through the website. Eligible participants were randomized between control (n=240) and intervention (n=250) groups. The well-being mobile app provides an 8-week program with 4 classes per week (including a brief theoretical portion and a 15-min guided practice). The active control app also provided 4 assessments per week that encouraged participants to self-observe how they were feeling for 20 min. We also used the app to conduct Web-based questionnaires (10-item Perceived Stress Scale and 5-item World Health Organization Well-Being Index) and ask specific questions to assess subjective levels of stress and well-being at baseline (*t*_1_), midintervention (*t*_4_=4 weeks after *t*_1_) and postintervention (*t*_8_=8 weeks after *t*_1_). Both apps were fully automated without any human involvement. Outcomes from the control and intervention conditions at the 3 time points were analyzed using a repeated measures analysis of variance.

**Results:**

Among the randomized participants (n=490), 185 participants were excluded at the 4-week follow-up and another 79 at the 8-week follow-up because of noncompliance with the experimental protocol. Participants who did not complete *t*_4_ and *t*_8_ assessments were equally distributed between groups (*t*_4_: control group=34.6% [83/240] and intervention group=40.8% [102/250]; *P*=.16; *t*_8_: control group=29.9% [47/157] and intervention group=21.6% [32/148]; *P*=.10). Both groups showed a significant increase in general well-being as a function of time (*F*_2,426_=5.27; *P*=.006), but only the intervention group presented a significant increase in work-related well-being (*F*_2,426_=8.92; *P*<.001), as well as a significant reduction in work-related and overall stress (*F*_2,426_=5.50; *P*=.004 and *F*_2,426_=8.59; *P*<.001, respectively).

**Conclusions:**

The well-being mobile app was effective in reducing employee stress and improving well-being.

**Trial Registration:**

Clinicaltrials.gov NCT02637414; https://clinicaltrials.gov/ct2/show/NCT02637414.

## Introduction

### Women's Mental Health

Over the last few decades, studies have been investigating the impact of occupational stress complaints on workers’ mental health [1,2]. Currently, there is a pressing need for a greater understanding of gender as a social determinant of health in the work context [3]. According to the American Psychological Association’s report *Stress in America: The State of Our Nation* [4], women consistently report higher stress levels than men, are more likely to say they experience symptoms of stress, and have difficulty dealing with it. Several studies indicate that work-related factors may affect women and men differently and suggest that women may be disproportionately affected because of work and family roles [5,6]. Research from the World Economic Forum’s Gender Gap study showed that women work nearly an hour longer than men every day, when both paid and unpaid tasks are considered [7]. Therefore, these findings indicate that working women may experience more stress than men and that the sources of stress are related to the expected and actual roles of women in society. Considering the increasing numbers of women in the workforce, it is critical that more resources be directed toward reducing the negative effects of work stress on women’s health and well-being.

### Meditation and Positive Psychology

Some very successful programs in stress management are based on the principle of Mindfulness—a metacognitive ability, defined by Jon Kabat-Zinn as “the awareness that emerges through paying attention on purpose, in the present moment, and nonjudgmentally to the unfolding experience moment to moment” [8]. The mechanism of action for mindfulness may be explained through a set of 4 interacting components [9]: (1) attention regulation (sustained attention, with returned attention on the main object of focus upon distraction), (2) body awareness (focused attention on subtle bodily sensations to enhance attunement with one’s body), (3) emotional regulation (practice of nonjudgmental awareness of one’s emotional responses in the moment), and (4) change in self-perspective (detachment from the view of an unchanging self). Although research exploring the effectiveness of online mindfulness-based interventions (MBIs) is still in its infancy, a recent meta-analysis concluded that there is emerging evidence that online MBIs have the potential to improve mental health outcomes, most notably stress [10].

Although the mental health care system has traditionally focused more on treatment of mental disorders than on prevention, it is recognized that mental health is more than just the absence of mental illness. Positive psychology is the study of well-being, engagement, and optimal functioning, fitting well with the World Health Organization’s (WHO) definition of mental health: “a state of well-being in which the individual realizes his or her own abilities, can cope with the normal stresses of life, can work productively and fruitfully, and is able to make a contribution to his or her community” [11]. The PERMA model proposed by Seligman [12] stipulates that happiness and psychological well-being is made up of 5 core components: positive emotions, engagement (similar to the concept of mindfulness), positive relationships, meaning, and accomplishments. Well-being has been shown to reduce the risk of developing mental health alterations or disorders [13,14]. Results from a meta-analysis of 51 positive psychology interventions with more than 4000 individuals revealed that such interventions do indeed significantly enhance well-being [15]. A number of studies conducted with adult [16] and younger [17] populations have shown improvements in well-being through the use of online positive psychology interventions. Technology-based self-care programs allow individuals to circumvent some of the limitations associated with traditional support methods, such as time, resources, flexibility, accessibility, and availability [18]. Indeed, this is in line with WHO directives recommending “the promotion of self-care, for instance, through the use of electronic and mobile health technologies” [19].

### Mobile Health

Mobile health (mHealth), which promotes the use of wireless technologies in health care, is one of the fastest growing fields within electronic health (eHealth) [20]. According to the *Digital 2019 Q2 Global Digital Statshot* report, 5.11 billion people own a mobile phone, which represents 66% of the world’s population [21]. About internet and mobile use in Brazil, the recently launched *Digital in 2019* report [22] showed that more than 149 million out of the country’s nearly 212 million inhabitants are active internet users and that there are 215.2 million mobile connections in Brazil, which represents a penetration of 102%. Moreover, the report stated that 66% of all Brazilians are mobile internet users and that there is an average use of 34 mobile apps per month per smartphone in the country. According to the IQVIA Institute, there are currently more than 318,000 health apps worldwide, nearly double the number of apps available in 2015, with more than 200 new health apps being added to app stores every day [23]. Even with this substantial increase over the previous report [24], consumer Digital Health apps targeting wellness management still account for the majority of health apps (60%), and mental health remains the largest focus for disease-specific mobile apps (28%) [23].

The use of a mobile device makes it possible to intervene and interact with the participants within the context (ie, their work environment) and during moments of their daily life, which is a form of intervention called ecological momentary intervention (EMI). The most reliable method for investigating real-world emotion is experience sampling, which involves contacting people as they engage in their everyday activities and asking them to report their thoughts, feelings, and actions at that moment [25]. Therefore, app-based EMIs offer a versatile, multifaceted, and interactive way of promoting training, mindfulness, self-awareness, motivation, and environmental awareness within the context of everyday life. The near ubiquitous use of smartphone apps provides a vehicle for making EMI a widespread and effective way of promoting positive change in a large nonclinical, nontherapeutic population.

Although smartphones and mobile apps seem to be ideal tools in many aspects for providing instantly accessible interventions to promote health, the content of these apps must be based on systematic research, as without such content, any observed effects could be placebo or even harmful. Even though the availability and use of mental health mobile apps have grown exponentially in recent years, few have been thoroughly tested to provide robust scientific evidence regarding their efficacy in modulating behavior or promoting health [26-28].

### Objectives

From these considerations, we developed 2 apps and hypothesized that a well-being mobile app, composed of an 8-week program with 4 lessons per week—based on relaxation training, breathing techniques, guided meditation, and positive psychology principles—could be effective in reducing stress and increasing well-being among working women when compared with an active control app designed to encourage self-observation and evaluation of subjective levels of stress and well-being, for the same period and weekly frequency. Here, we report the results of a randomized controlled trial in which we compared a well-being mobile app and an active control app on improving well-being and reducing stress in a group of women working in a private hospital in São Paulo, Brazil. In short, the aim of this study was to evaluate whether it is possible to improve psychological health by promoting stress management and well-being using a mobile app.

## Methods

### Trial Design

The experimental design of this study and the format of the manuscript followed the Consolidated Standards of Reporting Trials (CONSORT) statement for reporting randomized controlled trials [29], its extension for nonpharmacological trials [30], and the CONSORT-EHEALTH checklist [31]. See Multimedia Appendix 1 for CONSORT-EHEALTH checklist (V 1.6.1). The trial was registered at ClinicalTrials.gov, number NCT02637414 (Flourishing App: Evaluation of the Effectiveness of a Well-being App for Mobile Devices), on December 11, 2015. This study was a 2-arm randomized controlled trial conducted in Brazil. Participants were randomized using a 1:1 allocation ratio to 1 of the 2 parallel groups: (1) well-being mobile app based on relaxation, breathing, meditation, and positive psychology principles and (2) control app, containing only instructions to self-observation for 20 min and recording of subjective levels of stress and well-being. Both apps were developed in Brazilian Portuguese language and were fully automated without any human involvement. Longitudinal assessments were conducted at baseline, midintervention (4 weeks after baseline), and postintervention (8 weeks after baseline).

### Participants

We conducted this study at a large private tertiary care hospital in São Paulo, Brazil, from June 2016 to May 2017. We used mailing lists and intranet ads to invite hospital staff to participate in the study. Individuals were included if they met the following inclusion criteria: women aged between 20 and 60 years who had completed high school, owned a mobile device with either an iOS or Android operating system, and were available to participate in the 8-week training program. Potential participants first provided demographic data through an initial registration form, and those who were selected were then randomly assigned to 1 of the 2 groups (control or intervention). After that, they were given access to 1 of the 2 apps and a tutorial with instructions for its use. Excluded participants were informed via phone or email.

### Interventions

#### Intervention Condition

The well-being mobile app consists of an 8-week program divided into 2 4-week modules. Participants had to attend 4 classes per week; if they did not meet this deadline, they were given 4 more days to complete the classes, after which the app was blocked. Each class contained a brief theoretical portion and a 15-min guided practice. Twice a week, participants were asked to write down 1 good thing they had experienced and 1 good deed they had performed in a gratitude journal. They were told to do the activities whenever and wherever it felt most appropriate. Some screenshots of this app are provided in Multimedia Appendix 2.

The well-being mobile app was designed to handle psychological stress based on relaxation training, breathing techniques, meditation (such as mindfulness, loving, kindness, and empathetic joy), and positive psychology principles. The major aim of relaxation training and the use of breathing techniques is to reduce chronic stress and enhance well-being by eliciting a relaxation response, defined as a physical state characterized by decreased arousal of the sympathetic nervous system and the opposite of the body’s stress response to perceived threats [32]. An emerging approach to increase awareness and respond skillfully to mental processes that contribute to emotional distress and maladaptive behavior is based on mindfulness meditation, a process of maintaining a moment-by-moment awareness of our thoughts, feelings, bodily sensations, and surrounding environment, without judging them [8]. Finally, positive psychology activities that aim to cultivate positive feelings, behaviors, or cognitions such as practicing optimistic thinking, expressing gratitude, practicing kindness, and replaying positive experiences are another promising approach to increase psychological well-being [33,34].

During the first 2 weeks, the theoretical content dealt with physical and psychological consequences of chronic stress and the use of breathing techniques as a way to reduce it [32,35]. Guided practice was a technique known as body scan, which involves a gradual sweeping of attention through the entire body, focusing noncritically on any sensation or feeling in body regions and using periodic suggestions of breath awareness and relaxation [36]. The third and fourth weeks focused on mindful breathing to cultivate present moment awareness and meditation, respectively [8]. Guided practice during these 2 weeks involved meditation based on breath counting, using the breath as an anchor when the mind starts to wander [37]. The fifth week presented information on cultivating positive emotions and its impact on one’s health, life, and interpersonal relationships [12]. It included a guided loving kindness meditation, which is a method for developing the heartfelt yearning that all may find happiness [38]. During the sixth week, the theoretical content focused on the influence of positive and negative thoughts on different emotional states [12], and the guided exercise trained focusing attention and increasing awareness of one’s own repertoire of emotions, thoughts, and sensations [37]. During the seventh (second to last) week, the concept of empathy was explored [12], and the practice was a guided empathetic joy meditation aimed to train the manifestation of contentment through one’s own and others’ happiness [38]. The focus of the final week was gratitude and the different ways to cultivate it in daily routine [12], and the guided practice was mindfulness of breathing to focus on what participants were grateful for in the present moment [36]. Finally, all the practices taught during the stress management and wellness promotion program were reviewed. Toward the end of the intervention, participants were encouraged to keep practicing and to apply what they learned to everyday activities.

#### Control Condition

The control app had the same features as the well-being mobile app, including a menu, a tutorial, a profile page, evaluations, pop-up messages, and push notifications. Some screenshots of this app are provided in Multimedia Appendix 3. This active control condition followed the same period as the intervention. As in the intervention condition, participants had to answer 4 assessments per week. Each assessment was composed of a pre- and postevaluation separated by a 20-min interval. During this period, the control group received the exactly following instruction “Within 20 minutes, you will be invited to respond to these questions again. During this period, try to observe yourself and see how you are feeling,” whereas the intervention group carried out the proposed activities in the well-being mobile app. Due to this, we decided to call the active control condition *Monitoring of Perceptions*.

This approach was chosen as an active control for involving a mindfulness training (being aware of their perceptions) and is significantly better than the waiting list condition, which does not consider nonspecific effects of training [39], such as received attention and demand characteristics, or the possibility of a *digital placebo effect* (ie, placebo-like effects seen from mobile health interventions, such as smartphone apps) [40]. Recent technological advances have led to the design of well-being interventions that include adequate experimental controls [41].

#### Adherence Rates

The adherence rates were collected for all participants by accessing their accounts. The steps of users’ navigation flow in both apps were controlled by the following 3 steps: (1) completion of the preevaluation, (2) class (intervention condition) or monitoring of perceptions (control condition), and (3) completion of the postevaluation. We had a new record in the database every time the user completed any evaluation, and we considered an activity as concluded if the user filled the respective postevaluation form. Adherence was based on the number of classes or assessments that the participant concluded during the first 4 weeks and at the end of the 8-week period of the study. Moreover, 1 push notification per day and 2 emails per week were sent to participants to engage them.

### Outcomes: Data Collection

Primary outcome measures were taken at baseline (*t*_1_), midintervention (*t*_4_=4 weeks after *t*_1_), and postintervention (*t*_8_=8 weeks after *t*_1_). We assessed stress perception using the Brazilian Portuguese version of the 10-item perceived stress scale (PSS-10) [42,43] and subjective well-being with the Brazilian Portuguese version of the 5-item World Health Organization Well-Being Index (WHO-5) [44,45]. These Web-based questionnaires were applied using Google Forms through a link sent to participants via email. We also used an in-app questionnaire to assess subjective symptoms of stress and well-being at work and overall during the previous 30-day period. Moreover, within the app, secondary measures were gathered 4 times a week during 8 weeks, before and after the period of each class (intervention condition) or monitoring of perceptions (control condition) by assessing current subjective symptoms of stress and well-being.

### Primary Outcome Measures

#### 10-Item Perceived Stress Scale

This scale was developed by Cohen et al [46] to assess the degree to which individuals perceive their life situations as stressful. The perceived stress assessed by PSS corresponds to the issue of the cognitive appraisal process, when a “situation is appraised both as threatening or otherwise demanding and as taxing or exceeding the coping resources of the person.” In summary, the PSS items check how much respondents consider their lives unpredictable, uncontrollable, and overloaded; it also includes the number of items inquiring about current levels of experienced stress. PSS is a general scale as the content of its questions is not specific to any subpopulation or age group; however, it targets participants who have completed junior high school. The 10 questions address the frequency of participants’ feelings and thoughts about events and situations that occurred during the previous 30 days. A total of 6 questions are negative (1, 2, 3, 6, 9, and 10) and 4 are positive (4, 5, 7, and 8). Each question is rated on a 5-point Likert scale from 0 (never) to 4 (very often). To calculate the total score, the 4 positive items are reverse scored and summed across all scale items. Total scores may range from 0 to 40, and higher scores indicate higher levels of perceived stress. As PSS is not a diagnostic measure and there is no official cut-off available, recent studies [47,48] used 1 standard deviation (SD 6.2) above the mean PSS-10 of 15.3 in a large working population [49] as a cut-off value to choose participants with elevated stress levels (PSS-10≥22). Heber et al [47] also calculated the clinically significant change, according to the method developed by Jacobson and Truax [50], and determined that participants changed reliably if their PSS-10 score differed by more than ±5.16 points between assessments. Finally, Ebert et al [48] considered the symptom-free status, defined at PSS-10 <17.70, as the effect outcome for the cost-effectiveness analysis of their intervention. In our sample, the Cronbach alpha for the internal reliability of the PSS-10 was .82, which is similar to that observed in the original study (alpha=.78) [42] and in the Brazilian Portuguese version of the PSS-10 (alpha=.87) [43].

#### 5-Item World Health Organization Well-Being Index

WHO-5 is a short questionnaire consisting of 5 simple, noninvasive, and positively phrased items for measuring subjective well-being. Participants rate each of the 5 statements on a 6-point Likert scale, from 5 (all of the time) to 0 (at no time) to indicate how they felt during the last 2 weeks. The raw score theoretically ranges from 0 (absence of well-being) to 25 (maximal well-being), but it is recommended that one multiply the result by 4 to translate it to a percentage scale from 0 to 100, with higher scores meaning better subjective well-being. A score below 50% suggests poor emotional well-being [51], and the threshold for a clinically relevant change is considered to be 10% on standardized percentage scores [52]. A recent systematic review [52] showed that the WHO-5 is a helpful tool for clinical practice and research to evaluate well-being over time or to compare well-being between groups. The authors recommend using the general population’s mean score as a reference when using the WHO-5 as an outcome measure in clinical trials. In our sample, the Cronbach alpha was .88, which indicates a good internal validity. Similar internal consistency was found in the Brazilian Portuguese version of the WHO-5 (alpha=.83) [45].

#### Subjective Symptoms of Stress and Well-Being During the Last Month

We assessed subjective symptoms of stress and well-being at work and in general during the previous 30 days using a sliding percentage scale from 0 (absent) to 100 (maximal). Participants answered the following 4 simple questions: (1) What was your level of stress at work during the last month? (2) In general, what was your level of stress during the last month? (3) What was your level of well-being at work during the last month? and (4) In general, what was your level of well-being during the last month?

### Secondary Outcome Measures

#### Subjective Symptoms of Stress and Well-Being at the Moment

We evaluated subjective symptoms of stress and well-being before and after the period of each class (intervention condition) or monitoring of perceptions (control condition) using a sliding percentage scale from 0 (absent) to 100 (maximal). At this time, 2 simple questions were presented within the app: (1) What is your level of stress at this moment? and (2) What is your level of well-being at this moment?

#### Sample Size

The sample size was calculated according to a confidence interval of 0.95, a sampling error of 0.05, and a power effect of 0.8. From these data, a sample size calculation was conducted, and a minimum requirement of 100 participants in each group was determined.

#### Recruitment, Randomization, and Blinding

Potential participants were invited to participate by email and hospital intranet ads. Candidates were asked to access the study’s website [53], read the information package, and fill out an online registration form. Participants who met the inclusion criteria were randomly assigned to 1 of the 2 trial arms. A research assistant with no clinical involvement in the trial used the Microsoft Excel RANDBETWEEN function to randomly assign either 0 or 1 to each participant, corresponding to each experimental arm. The same research assistant generated and distributed unique access codes. The participants were aware of their allocated study arm, whereas outcome assessors and data analysts remained blind to group allocations until the completion of the study.

#### Statistical Analysis

Postintervention analyses involved a modified intention-to-treat design excluding participants who did not adequately adhere to the protocol. We developed the app to be a stress management and wellness promotion training program. As courses or trainings usually require a minimum frequency of 75% so that the participant does not fail because of absences, we decided to use this cut-off, even if it was somewhat stringent for mobile apps. Thus, only volunteers who completed 12 of the 16 activities during the first 4 weeks, completing 75% of module I, were included in the sociodemographic analysis, as well as the analysis comparing baseline with mid-intervention scores. Likewise, only volunteers who completed 23 of the 31 activities of the following 4 weeks, completing 75% of module II, were included in the analyses.

Statistical analyses were conducted using IBM SPSS Statistics for Windows, version 24.0 (IBM Corp). Descriptive analyses were used for sociodemographic data to report absolute and relative frequencies as well as means, standard deviations, and medians. The Kolmogorov-Smirnov test was used to evaluate data normality. The Pearson chi-square test was used to compare categorical variables between groups at baseline, whereas Student *t* tests (or the nonparametric Wilcoxon-Mann-Whitney test, WMW) were used to compare the continuous variables. A repeated measures analysis of variance (ANOVA) was used to evaluate primary outcomes, considering 2 conditions (control and intervention) and 3 distinct periods (baseline, midintervention, and postintervention). The time × group interaction effects were assessed to investigate differences regarding the magnitude of change on dependent variables between the experimental and control groups. For the secondary outcome measures (ie, assessments made before and after each activity), we used the WMW test to compare stress and well-being level variations (∆SL and ∆WBL, respectively) between groups. The significance threshold was set at .05.

#### Ethics and Consent

Concerning data confidentiality, special efforts were made to secure online data collection and storage. Data collected using Google Forms were only accessed by the study’s first author. When these data were downloaded, other authors had access to the relevant data files. Data collected via the project website and within the app were stored on Amazon Relational Database Service. The platform used was an AWS Elastic Beanstalk, and its security criteria are available on the Web page [54]. The server stores data in an unencrypted database, but access is granted through authentication by inserting a log-in, password, and security token. All data were saved in both .xlsx and .sav formats. These files were stored in a password-protected folder on a secure server and were only available to the research team. In accordance with the Hospital Israelita Albert Einstein data retention policy, these data will be retained for 5 years after the end of the study and anonymized by replacing participant names with subject ID numbers.

The study protocol and consent procedure were approved by the Ethics Committee of the Instituto Israelita de Ensino e Pesquisa Albert Einstein—Brazil (nº 1.469.020). Study participants were given adequate information before agreeing to participate and freely providing informed consent. Documentation of written consent was not mandatory for this study because an online consent form was used (available on the study’s website) and participants could only access the registration form if they accepted and signed the consent form [53]. It was clearly communicated that their participation was voluntary and without monetary compensation. Participants were reminded that they were free to withdraw at any time, without penalty or need for explanation, and that their data would be stored securely and anonymously. Control participants were offered access to the well-being mobile app upon study completion. Although there were no reported risks associated with the stress management and wellness promotion program or similar online interventions, the questionnaires and activities could cause discomfort for some people. Given that there were no adverse effects associated with using the app, we had no reason to discontinue the intervention for individuals who chose to continue.

## Results

### Participant Flow

**Figure 1:**
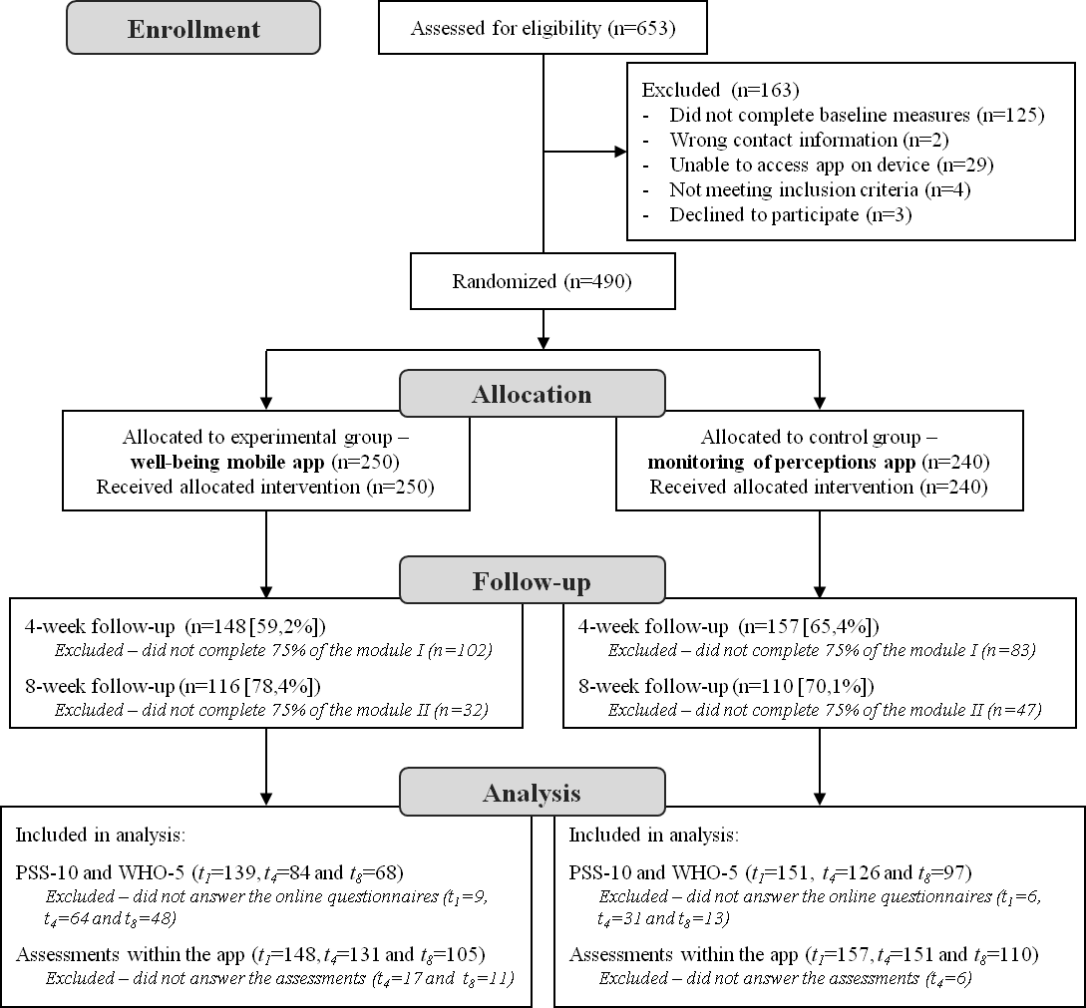
Consolidated Standards of Reporting Trials flow diagram. PSS-10: 10-item Perceived Stress Scale; WHO-5: 5-item World Health Organization Well-Being Index.

In September 2016, 653 individuals were enrolled in the study through the website and were then screened for eligibility. Among these potential participants, 163 were excluded (because they did not complete baseline measures or were unable to access the app) and the remaining 490 were randomized (see Figure 1 for a flow diagram). We excluded an additional 185 participants at the 4-week follow-up and another 79 at the 8-week follow-up because of noncompliance with the experimental protocol (ie, completed less than 75% of each module). Participants who did not complete *t_4_* and *t_8_* assessments were equally distributed between groups (*t*_4_: control group=34.6% [83/240] and intervention group=40.8% [102/250]; X^2^_1_=2.0; *P*=.16; effect size for chi-square test ϕ=0.064; *t*_8_: control group=29.9% [47/157] and intervention group=21.6% [32/148]; X^2^_1_=2.7; *P*=.10; ϕ=0.095).

### Baseline Data

**Figure 2:**
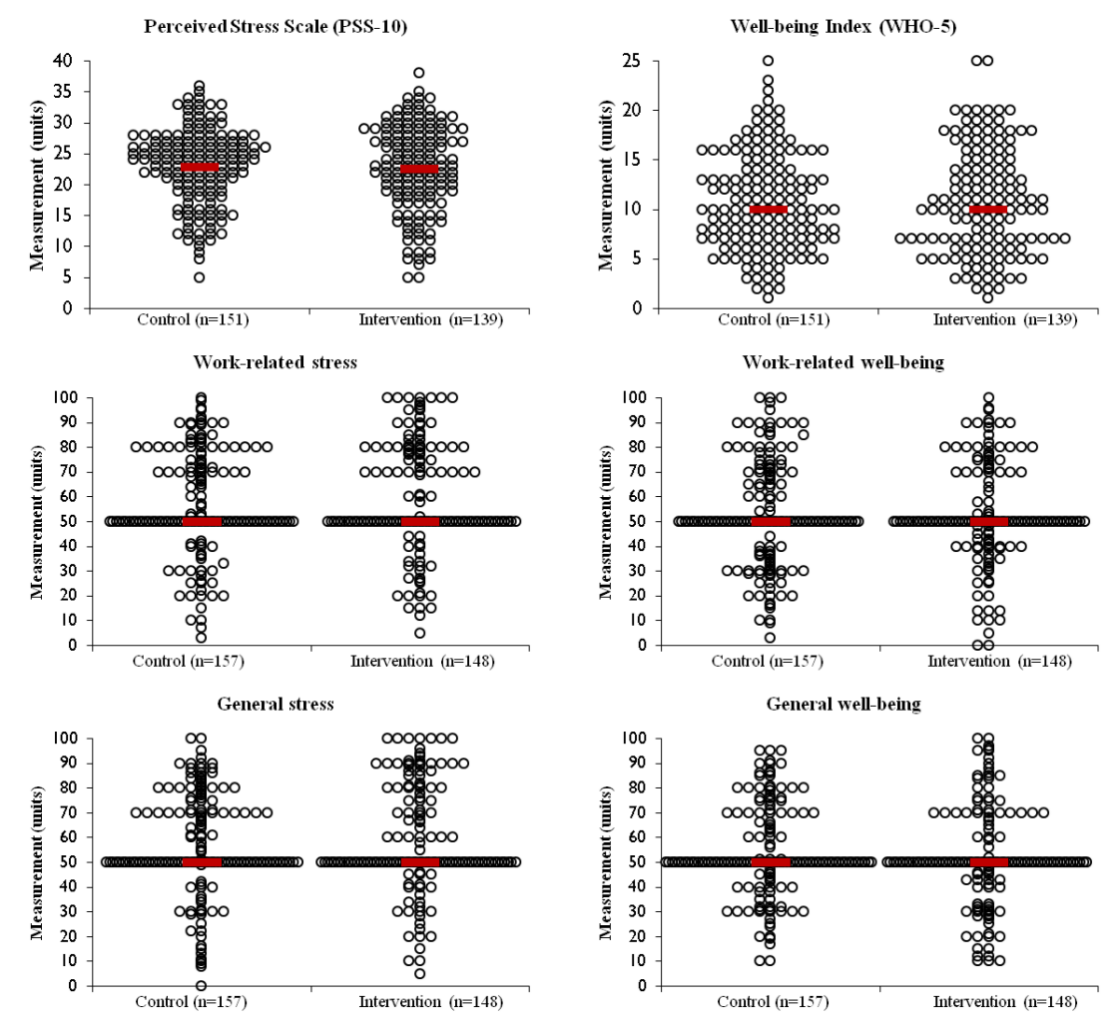
Baseline outcome score data by experimental group. Scatter plots showing bivariate analyses of 10-item Perceived Stress Scale (*t*_288_=0.414; *P*=.68; *d*=0.045), 5-item World Health Organization Well-Being Index (*U*=10461; *P*=.96; *r*=−0.002), work-related stress (*U*=11078; *P*=.47; *r*=−0.041), general stress (*U*=11274; *P*=.64; *r*=−0.026), work-related well-being (*U*=11487; *P*=.86; *r*=−0.010), and general well-being (*U*=11060; *P*=.45; *r*=−0.043) for the control and intervention groups. Open circles show data for each participant. Red lines show group medians, except for PSS-10, which shows group means. PSS-10: 10-item Perceived Stress Scale; WHO-5: 5-item World Health Organization Well-Being Index.

Table 1 and Figure 2 show the baseline sociodemographic and outcome score data for both groups, respectively. Overall, the mean age of the participants was 34.6 (SD 7.62) years; 53% (n=161) were married or lived with a domestic partner; 45% (n=137) had a graduate degree; and 65% (n=197) were professionals other than nurses, physicians, or professionals in leadership positions. On average, PSS-10 scores were 22.73 (SD 6.69), and WHO-5 scores were 10.75 (SD 5.14). As seen in Table 1 and Figure 2, baseline characteristics were equivalent. See Multimedia Appendix 4 for mean (SD) and median (p25-p75) values.

**Table 1:** Baseline sociodemographic data by experimental group.

Characteristics^a^		Control (n=157)	Intervention (n=148)	*P* value
Age (years), mean (SD)		33.8 (7.47)	35.4 (7.73)	.07
Children, median (p25-p75)^b^		0 (0-1)	1 (0-1)	.75
**Profession, n (%)**				.77
	Assistant	0 (0.0)	1 (0.7)	—^c^
	Analyst	1 (0.6)	1 (0.7)	—
	Leadership coordinator	10 (6.4)	8 (5.4)	—
	Leadership manager	1 (0.6)	0 (0.0)	—
	Professional	105 (66.9)	92 (62.2)	—
	Nursing	33 (21.0)	39 (26.4)	—
	Physician	7 (4.5)	7 (4.7)	—
**Marital status, n (%)**				.68
	Single	61 (38.9)	52 (35.1)	—
	Married/cohabiting	78 (49.7)	83 (56.1)	—
	Separated/divorced	16 (10.2)	12 (8.1)	—
	Others	2 (1.3)	1 (0.7)	—
**Educational level, n (%)**				.40
	Complete high school	30 (19.1)	28 (18.9)	—
	Incomplete higher education	20 (12.7)	25 (16.9)	—
	Complete higher education	39 (24.8)	26 (17.6)	—
	Postgraduate degree	68 (43.3)	69 (46.6)	—

^a^Statistical test results comparing age (*t*_303_=−1.803; Cohen *d*=0.21), number of children (Mann-Whitney *U* test, *U*=11388; effect size correlation for Mann-Whitney *U* test, *r*=−0.018), profession (*X̄*^2^_6_=3.3; effect size Cramer *V*=0.104), marital status (*X̄*^2^_3_=1.5; *V*=0.070) and educational level (*X̄*^2^_3_=2.9; *V*=0.099) for the control and intervention groups.

^b^Data are presented as medians (25th-75th percentile).

^c^Not applicable.

### Outcomes

#### Preintervention × Midintervention Analysis

**Figure 3:**
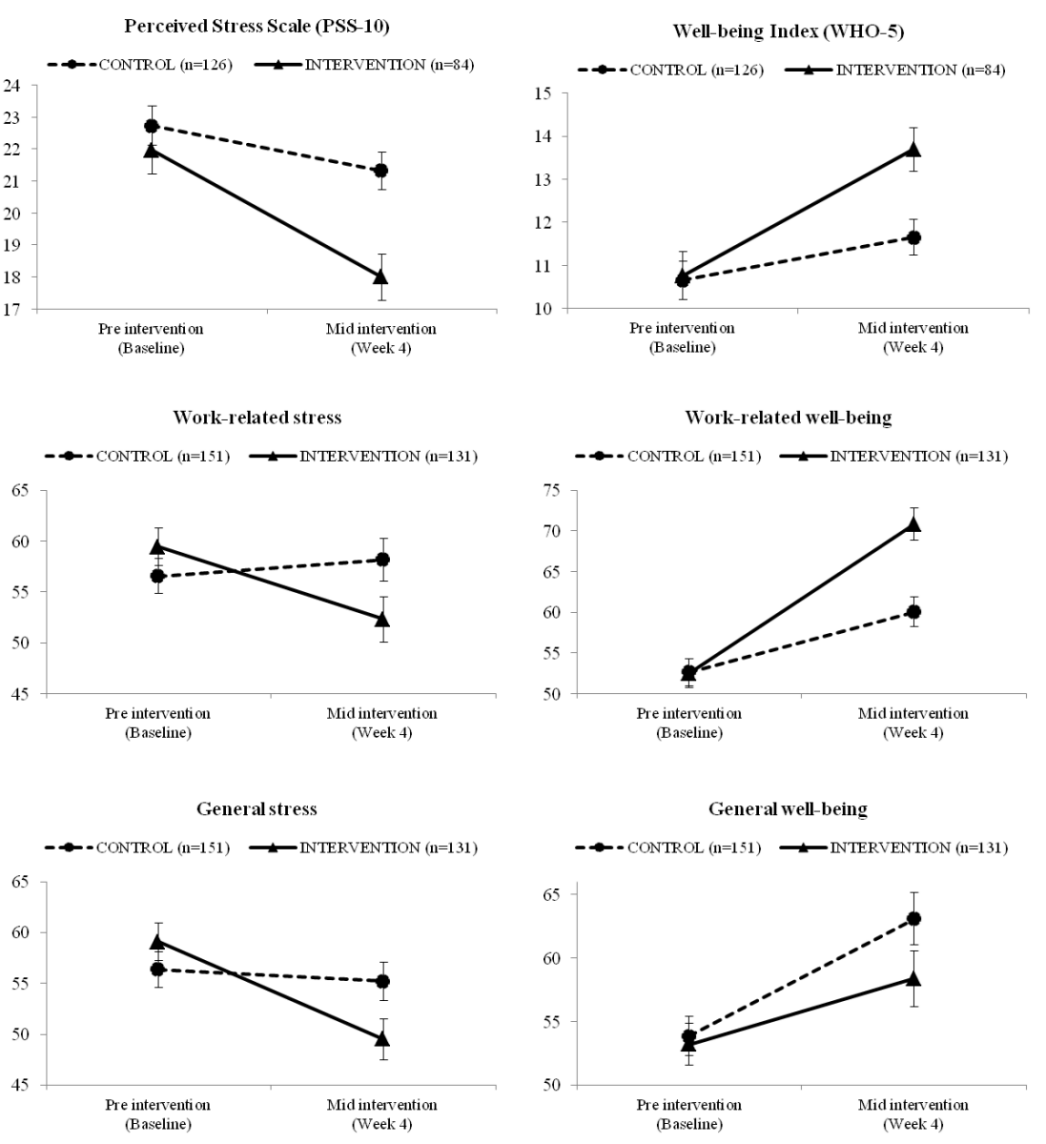
Plot means with standard error bars showing all outcome data for the control (dashed black line) and intervention (solid black line) groups at pre intervention and mid intervention. Time × group interaction at 10-item Perceived Stress Scale (*F*_1,208_=9.48; *P*=.002; η_p_^2^=.044), 5-item World Health Organization Well-Being Index (*F*_1,208_=8.54; *P*=.004; η_p_^2^=.168), work-related stress (*F*_1,208_=6.34; *P*=.01; η_p_^2^=.022), work-related well-being (*F*_1,208_=12.1; *P*=.001; η_p_^2^=.041), general stress (*F*_1,208_=6.46; *P*=.01; η_p_^2^=.023), and general well-being (*F*_1,208_=1.48; *P*=.22; η_p_^2^=.005). PSS-10: 10-item Perceived Stress Scale; WHO-5: 5-item World Health Organization Well-Being Index.

Efficacy results from the first 4 weeks of intervention, using a per-protocol approach, are presented in Figure 3. See Multimedia Appendix 5 for descriptive data related to repeated measures analysis of variance. Across groups, PSS-10 scores significantly decreased from preintervention (sample mean, 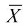
¯=22.35; SE=0.48) to midintervention (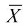
=19.65; SE=0.47; *F*_1,208_=41.9; *P*<.001; *η_p_*^2^=0.168). A time × group interaction (*F*_1,208_=9.48; *P*=.002; *η_p_*^2^=0.044) indicated that the intervention group experienced significant decreases in perceived stress compared with the control condition.

WHO-5 scores increased for all participants as a function of time (*F*_1,208_=35.4; *P*<.001; *η_p_*^2^=0.146). A time × group interaction was also observed (*F*_1,208_=8.54; *P*=.004; *η_p_*^2^=0.168) with participants in the intervention condition showing a greater increase on the well-being index from preintervention to midintervention than participants in the control group.

Work-related stress did not change as a function of time alone (*F*_1, 280_=2.54; *P*=.11; *η_p_*^2^=0.009) but changed as a function of time × group (*F*_1,280_=6.34; *P*=.01; *η_p_*^2^=0.022), such that people in the intervention condition reported greater improvements in work-related stress from preintervention to midintervention compared with participants in the control group.

General stress showed a significant time effect (*F*_1,280_=10.4; *P*=.001; *η_p_*^2^=0.036), with lower general stress ratings from preintervention (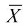
¯=57.71; SE=1.26) to midintervention (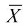
¯=52.37; SE=1.38). A significant time × group interaction emerged (*F*_1,280_=6.46; *P*=.01; *η_p_*^2^=0.023), such that participants in the intervention condition experienced greater reductions in general stress than those in the control group.

Work-related well-being significantly increased across participants from preintervention (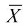
¯=52.57; SE=1.22) to midintervention (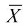
¯=65.43; SE=1.34; *F*_1,280_=67.2; *P*<.001; *η_p_*^2^=0.194), and there was a significant time × group interaction (*F*_1,280_=12.1; *P*=.001; *η_p_*^2^=0.041), showing a greater benefit for individuals in the intervention group.

General well-being increased as a function of time from preintervention (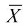
¯=53.53; SE=1.12) to midintervention (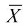
¯=60.72; SE=1.52; *F*_1,280_=18.5; *P*<.001; *η_p_*^2^=0.062), but no time × group interaction was observed.

#### Preintervention × Midintervention × Postintervention Analysis

**Figure 4:**
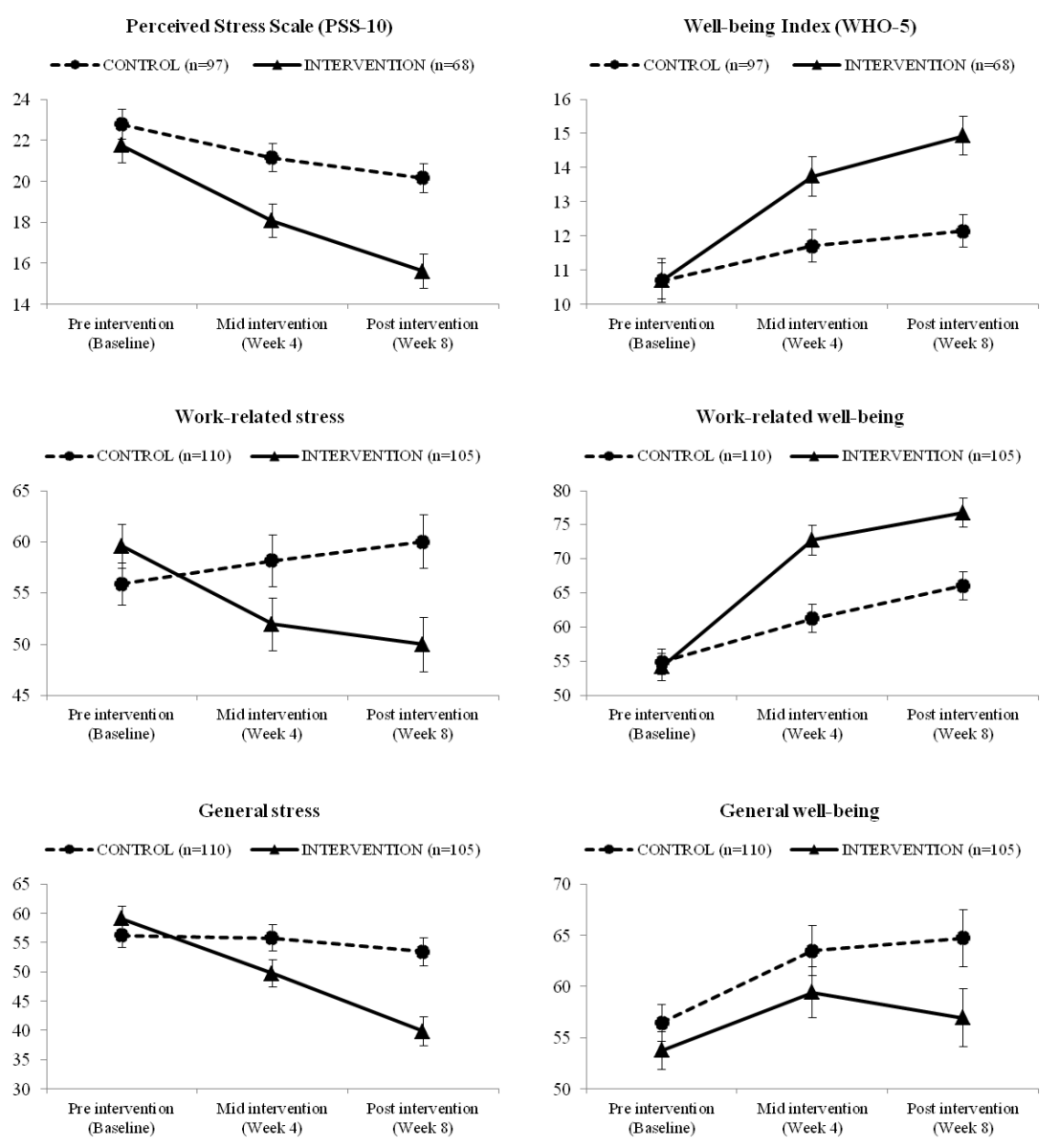
Plot means with standard error bars showing all outcome data for the control (dashed black line) and intervention (solid black line) groups at preintervention, midintervention, and postintervention. A time × group interaction was observed at 10-item Perceived Stress Scale (*F*_2,326_=7.19; *P*=.001; *η*_p_^2^=.042), 5-item World Health Organization Well-Being Index (*F*_2,326_=7.97; *P*<.001; *η*_p_^2^=.047), work-related stress (*F*_2,426_=5.50; *P*=.004; *η*_p_^2^=.025), work-related well-being (*F*_2,426_=8.92; *P*<.001; *η*_p_^2^=.040), general stress (*F*_2,426_=8.59; *P*<.001; *η*_p_^2^=.039), and general well-being (*F*_2,426_=0.74; *P*=.47; *η*_p_^2^=.003). PSS-10: 10-item Perceived Stress Scale; WHO-5: 5-item World Health Organization Well-Being Index.

Intervention efficacy results using a per-protocol approach are shown in Figure 4. See Multimedia Appendix 6 for descriptive data related to repeated measures analysis of variance. Analysis of PSS-10 scores revealed a time effect (*F*_2,326_=45.0; *P*<.001; *η_p_*^2^=0.216), and pairwise comparisons indicated significant declines in PSS-10 scores from preintervention (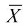
¯=22.27; SE=0.56) to midintervention (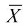
¯=19.60; SE=0.53), and again from midintervention to postintervention (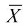
¯=17.88; SE=0.54). A time × group interaction (*F*_2,326_=7.19; *P*=.001; *η_p_*^2^=0.042) indicated that the intervention group produced significant decreases in perceived stress when compared with the control group.

WHO-5 scores increased across all participants as a function of time (*F*_2,326_=33.4; *P*<.001; *η_p_*^2^=0.170), and pairwise comparisons indicated significant increases in WHO-5 scores from preintervention (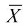
¯=10.69; SE=0.41) to midintervention (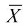
¯=12.72; SE=0.37), and again from midintervention to postintervention (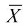
¯=13.53; SE=0.37). A time × group interaction was also observed (*F*_2,326_=7.97; *P*<.001; *η_p_*^2^=0.047), with participants in the intervention condition showing a greater increase in well-being than participants in the control group.

Work-related stress did not change as a function of time alone (*F*_2,426_=1.09; *P*=.34; *η_p_*^2^=0.005) but changed as a function of time × group (*F*_2,426_=5.50; *P*=.004; *η_p_*^2^=0.025), such that people in the intervention condition reported greater improvements in work-related stress compared with those in the control group.

General stress showed a significant time effect (*F*_2,426_=15.3; *P*<.001; *η_p_*^2^=0.067), and pairwise comparisons indicated significant decreases in general stress ratings from preintervention (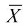
¯=57.63; SE=1.49) to midintervention (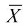
=52.72; SE=1.61), and again from midintervention to postintervention (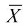
¯=46.60; SE=1.73). A significant time × group interaction was observed (*F*_2,426_=8.59; *P*<.001; *η_p_*^2^=0.039), such that participants in the intervention condition experienced greater reductions in general stress than those in the control condition.

Work-related well-being significantly increased across participants from preintervention (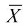
¯=54.55; SE=1.36) to midintervention (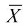
¯=67.03; SE=1.51) and again from midintervention to postintervention (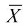
=71.43; SE=1.47; *F*_2,426_=58.5; *P*<.001; *η_p_*^2^=0.215), and there was a significant time × group interaction (*F*_2,426_=8.92; *P*<.001; *η_p_*^2^=0.040), showing a greater benefit for individuals in the intervention group.

General well-being increased as a function of time (*F*_2,426_=5.27; *P*=.006; *η_p_*^2^=0.024), and pairwise comparisons indicated significant increases in general well-being ratings from preintervention (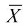
¯=55.08; SE=1.27) to midintervention (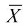
=61.45; SE=1.75) and to postintervention (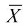
¯=60.82; SE=1.98). Unlike the other variables analyzed thus far, no differences were observed between mid- and postintervention measurements for general well-being. There was also no time × group interaction.

#### Changes in Subjective Symptoms of Stress and Well-Being After the Proposed Daily Activities

For secondary outcome evaluations, we compared stress and well-being level variations (∆SL and ∆WBL, respectively) between groups after completion of 75% of the classes in module I. As expected, participants using the well-being mobile app reported a significantly greater reduction in stress levels (Table 2) and significantly greater increases in well-being levels (Table 3) than those using the active control app.

**Table 2:** Between-group comparisons of stress level variations after each class.

Class number	Control		Intervention		Significance		
	n	Median (p25-p75)^a^	n	Median (p25-p75)	*U* ^b^	*P* value	*r* ^c^
Class 1	126	0.00 (−15.0-4.25)	148	−15.0 (−30.0-0.00)	5897	<.001	−0.309
Class 2	90	0.00 (−9.00-9.25)	148	−12.5 (−27.7-0.00)	3675	<.001	−0.377
Class 3	81	0.00 (−20.0-4.00)	148	−11.5 (−26.7-0.00)	4671	.006	−0.183
Class 4	87	0.00 (−13.0-3.00)	148	−9.50 (−21.0-0.00)	4667	<.001	−0.231
Class 5	83	0.00 (−9.00-5.00)	148	−9.00 (−26.0-0.00)	3352	<.001	−0.378
Class 6	87	0.00 (−13.0-0.00 )	148	−10.0 (−30.7-0.00)	4957	.003	−0.193
Class 7	88	0.00 (−6.00-0.75)	148	−8.00 (−21.0-0.00)	3883	<.001	−0.340
Class 8	88	0.00 (−7.75-2.50)	148	−6.00 (−29.0-0.00)	4464	<.001	−0.264
Class 9	98	0.00 (−4.00-3.00)	148	−10.0 (−26.7-0.00)	3945	<.001	−0.389
Class 10	103	0.00 (−9.00-4.00)	148	−8.00 (−20.7-0.00)	5153	<.001	−0.278
Class 11	95	0.00 (−7.00-4.00)	148	−4.50 (−13.7-0.00)	4961	<.001	−0.251
Class 12	97	0.00 (−10.0-1.00)	148	−7.00 (−17.7-0.00)	5170	<.001	−0.238

^a^Data are presented as medians (25th-75th percentile).

^b^Mann-Whitney *U* test.

^c^Effect size correlation for Mann-Whitney *U* test.

**Table 3:** Between-group comparisons of well-being level variations after each class.

Class number	Control		Intervention		Significance		
	n	Median (p25-p75)^a^	n	Median (p25-p75)	*U* ^b^	*P* value	*r* ^c^
Class 1	126	1.50 (−5.25-20.0)	148	20.0 (5.25-40.0)	5108	<.001	−0.390
Class 2	90	0.00 (−2.00-10.0)	148	18.0 (4.00-30.7)	3165	<.001	−0.441
Class 3	81	3.00 (0.00-14.0)	148	13.5 (2.00-30.0)	3821	<.001	−0.300
Class 4	87	0.00 (−2.00-13.0)	148	13.0 (0.00-29.2)	4105	<.001	−0.303
Class 5	83	0.00 (−6.00-10.0)	148	11.0 (1.25-25.7)	3163	<.001	−0.404
Class 6	87	1.00 (0.00-10.0)	148	10.0 (0.00-29.7)	4335	<.001	−0.274
Class 7	88	0.00 (−1.00-5.00)	148	13.0 (1.00-29.0)	3057	<.001	−0.447
Class 8	88	0.00 (−1.75-5.75)	148	11.0 (0.00-29.2)	3678	<.001	−0.366
Class 9	98	0.00 (−2.00-10.0)	148	14.0 (2.00-26.7)	3876	<.001	−0.395
Class 10	103	0.00 (−5.00-9.00)	148	9.00 (0.25-18.7)	4635	<.001	−0.335
Class 11	95	0.00 (−5.00-2.00)	148	9.50 (0.00-21.0)	3389	<.001	−0.439
Class 12	97	0.00 (−2.00-9.00)	148	9.50 (0.00-20.0)	4541	<.001	−0.312

^a^Data are presented as medians (25th-75th percentile).

^b^Mann-Whitney *U* test.

^c^Effect size correlation for Mann-Whitney *U* test.

## Discussion

### Principal Findings

The primary aim of this study was to evaluate the effectiveness of a mobile app promoting stress management and well-being for working women. To this end, we conducted a 2-arm randomized controlled pragmatic trial with female employees at a private hospital using a long period program (8 week), a self-selected sample, and standardized questionnaires. The pragmatic trial is designed to test the effectiveness of an intervention in everyday life to maximize applicability and generalizability [55]. Groups were homogeneous regarding baseline characteristics, and although both groups showed a significant increase in general well-being, only the intervention group presented a significant increase on the well-being index (WHO-5 scores) and work-related well-being, as well as significant decreases in perceived stress (PSS-10 scores), work-related stress, and general stress. In addition, participants who used the well-being mobile app reported a significantly greater reduction in stress levels and a significant increase in well-being levels after each daily activity than participants who used the active control app. These results indicate that the well-being app was more effective at reducing employee stress and improving well-being levels.

One of our primary outcome measures was the PSS-10, the most widely used psychological instrument for measuring the perception of stress. Our results showed that participants from both groups were severely distressed at baseline scores (control [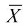
¯=22.9; SD 6.24] and intervention 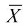
¯=22.6; SD 7.16]), and only the intervention group presented a clinically significant change in the PSS-10 score (−6.15 points, on average) at the end of the 8-week study period, reaching symptom-free status (PSS-10=15.6), as defined by Ebert et al [48].

The other primary outcome measure used in this study was the WHO-5, one of the most widely used questionnaires to assess subjective well-being. The WHO-5 baseline score for both groups was approximately 43%, which indicates reduced well-being. After 8 weeks, the control group had a WHO-5 increase of approximately 6%, whereas improvement for the intervention group was almost 17%. Therefore, the difference in WHO-5 scores between groups was approximately 11%, which is clinically significant. However, at the end of the 8-week study period, the participants in the intervention group still had mean WHO-5 values (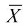
¯=14.93; 59.72%) below the general population reference level of 73.37% [45].

In the field of stress research, many studies use the WHO-5 to assess a wide variety of aspects including links between working conditions and well-being [56], the association between psychosocial working conditions and psychological well-being [57], as well as the association between workplace stress and well-being [58]. Our results corroborate the findings of Goa et al [58], who found that approximately 35% (n=977) of a total of 2796 employees presented low well-being scores (WHO-5 < 50) and that women, younger workers (<40 years), workers with high educational levels, and those with higher levels of job stress reported higher rates of poor mental health.

Several factors may contribute to the results obtained in this study. First, we applied an app-based EMI to intervene and interact with the participants in their work environment and during moments of their daily life. In addition, our system has been designed to ensure that participants respond to questions *in the moment*, that is, soon after being alerted to do so. This is a great advantage in relation to (1) surveys that require people to make retrospective and often generalized judgments, which tend to be affected by memory limitations and recall biases [59], and (2) laboratory experiments that do not occur within the context of a person's daily life, as we know that context can influence a person’s states and responses [60]. Self-monitoring has long been known to raise self-awareness and promote positive behavioral development under certain conditions [61,62]. Specifically, it has been theorized that being asked questions about one’s momentary states, experiences, behaviors, and/or thoughts close to the time and context of their occurrence may help one become more mindful of their occurrence, thereby providing opportunity for change [63]. Although it was only recorded as self-monitoring, participants in the control group reported that measuring stress and well-being levels before and after 20 min made them aware of changes in their physiological state (ie, they became more aware of their thought processes and more reflective). This could explain the increase in general well-being also observed in the active control group.

Second, our 8-week training program was built on the basis of 2 central approaches aimed at reducing stress and improving well-being. As described previously, meditation was one of those foundations. Studies involving smartphone delivery of mindfulness interventions focusing on workplace stress [64,65], well-being [41,66-68], and depression [69] showed comparable results to previous traditional interventions focused on the same outcome variables. The advantage of the smartphone interventions is that they are more rigorous because of instruction standardization across participants in the experimental group, the inclusion of active control materials participants expected to benefit from, and objective measures of adherence (provided within the app) rather than self-report. The other foundation was positive psychology principles. A recent meta-analysis revealed that 67% of positive psychology interventions are delivered in a self-help format, sometimes in conjunction with face-to-face instruction and support [34]. It is already known that a *shotgun* approach involving multiple and different positive exercises may be more effective than engaging in only 1 activity [70]. In our well-being mobile app, we used a combination of exercises and information to develop skills across different positive psychology domains, besides providing information about the benefits of increased well-being. From a public health perspective, self-help interventions can serve as cost-effective mental health promotion tools to reach large target groups that may not otherwise be reached [71].

Third, the theoretical basis of the intervention was confined to evidence-based components, consistent with the recommendations presented by Bakker et al [27] to create better and more rigorous mental health apps (MHapps). The chosen strategies were as follows: (1) focusing on nonclinical mental health, psychological well-being, and coping abilities, aiming to increase accessibility, enable preventive use, and reduce stigma, therefore avoiding the harmful effects of using mental illness labels [72]; (2) using self-monitoring and self-reflection—core features of many evidence-based psychological therapeutic techniques—to promote psychological growth and enable progress evaluations [73], with the advantage that MHapps make it possible for users to record self-monitoring data during their usual daily routines, while undergoing challenges or directly experiencing stressors [18]; (3) applying behavioral activation (ie, encourages individuals to engage in physiologically activating and psychologically rewarding activities) to boost self-efficacy, psychological well-being, and a repertoire of coping skills, since an app may promote self-discovery by encouraging an activity and then prompting reflection on the experience immediately after [74]; (4) presenting brief and passive psychoeducation to develop mental health literacy, in other words, to teach the participants about psychological processes underlying their distress and inform them about resources available to manage it [75]; (5) using real-time engagement to allow users to seek help for psychological challenges at the time they experience distress or soon after, thus opening new learning opportunities and applying coping strategies in ecologically valid contexts [76]; (6) promoting activities explicitly linked to specific mood problems to enhance understanding of cause-and-effect relationships between actions and emotions [77]; (7) using gamification—the use of “game-based mechanics, aesthetics and game thinking to engage people, motivate action, promote learning and solve problems” [78]—and intrinsic motivation to encourage app use via rewards and internal triggers, positive reinforcement, and behavioral conditioning, emergent approaches that may help counteract motivation problems and yield additional well-being outcomes; (8) providing reminders (email and push notifications) as external triggers for engagement, aiming to increase adherence and reduce dropout from self-help interventions [79]; and (9) conducting an experimental trial to establish the app’s efficacy before recommending it as an effective intervention.

Donker et al [26] systematically investigated the effectiveness of MHapps. Only 8 papers (describing 5 apps) were identified as providing scientific support for MHapps. Only 1 of these was a self-contained app, whereas the other 4 required input from a mental health professional. Unfortunately, none of these apps is currently available in app stores. These findings revealed the lack of experimental evidence for MHapps, of which hundreds are available. Despite the small number of studies included in their review, Donker et al [26] concluded that although apps have the potential to benefit those with poor mental health, further studies are needed to fully understand their usefulness and effectiveness. In summary, our results show that the intervention improved subjective symptoms of well-being and reduced stress through a wide array of evidence-based techniques that were delivered via a simple, enjoyable, intuitive, and interactive design. The simplicity of a program’s interface and ease of navigation significantly influence users’ perception of the quality of Web-based mental health interventions [80]. Our results corroborated the key outcomes observed by Coulon et al [81] that evidence-based, transparent, functional, and user-friendly apps may engage patients in effective stress reduction strategies.

Despite the proven efficacy of internet-based mobile-supported mental health interventions, high attrition rates are observed [66,82]. Nonadherence is a common issue in online psychological interventions and may reduce the effectiveness of an intervention [83]. A systematic review of attrition rates from internet-based intervention programs in which contact with a therapist was minimal reported an average dropout rate of 31% (range: 2%-83%) [84]. These data suggest that this trial had an acceptable attrition rate (54%) relative to other eHealth programs. Unfortunately, it is difficult to compare adherence levels of this trial with those of other internet-based stress management interventions because few studies thus far have reported this information. The percentage of participants in this study who completed both conditions (46%) was similar to available intervention completion rates (eg, 37%, [85]; 44%, [86]; and 88% [87]). The adherence rates of a meta-analysis of online MBIs varied between 35% and 92% [10].

Participants who adhered perceived the intervention as manageable and felt that they had learned useful strategies. On the other hand, many nonadherent participants declared that it was not easy to make time to do the exercises, although the self-help intervention helped them recognize the importance of self-regulating attention on their daily activities and finding personal space to relax. For this reason, it is critical to analyze the potential outcomes of traditional protocols delivered by new technologies. This critical investigation allows a better understanding of whether self-help mobile apps can be beneficial to users who are seeking well-being training to obtain better mental health.

Improving adherence is a high priority for internet interventions, as higher usage rates are associated with significant improvements in well-being [17]. Our mobile app used a *one size fits all* approach, which may not have been appropriate for a large group of people. Previous research indicates that providing support has a positive influence on adherence and enhances the effectiveness of online interventions [10,88]. Therefore, offering complementary instructor guidance to participants may potentially improve adherence and outcomes; however, instructor involvement is costly and may restrict the intervention’s scalability. These barriers may be partially overcome by using automated support. Automated support has been shown to improve intervention adherence and effectiveness [89].

The authors would like to highlight that this is a well-being and stress reduction educational app and that people suffering from mental disorders or whose symptoms are difficult to manage should seek support from a mental health professional. We would also like to emphasize that this app offers an initial experience in the field of self-care with introductory classes in meditation, relaxation, attention, and positive psychology topics. Although it may be an ideal tool for those who do not have time to attend regular classes, it is not intended to substitute the guidance of experienced/certified instructors for people interested in deepening their practice.

### Limitations

To interpret the results reported in this paper, some limitations should be taken into account. First, the main limitation of the study was that participants were not blinded, which may have increased bias. However, the designed study provided evidence of a pragmatic self-care intervention. Second, for feasibility reasons, the answers were self-reported. Third, we included only women and the volunteers self-selected into the trial, which limits the generalizability of the results. These limitations, however, should have minimal impact on the validity of the data, given the number of strengths of this study (eg, large sample size, use of an active control group, objective measures of intervention adherence, app developed for both Google’s Android and Apple’s iOS mobile operating systems).

### Future Research

Future research should aim to replicate the results of this trial and investigate variables that may affect outcome and adherence. Personalization and tailoring intervention to individual needs, from baseline stress and well-being levels, and interactive support could contribute to increased adherence and thereby to boost the effectiveness of the intervention. As all responses here were self-reported, integrating psychophysiological sensors with the apps and collecting biological measures, such as cortisol levels, would also strengthen the evidence of the beneficial effects of the well-being mobile app. Future studies are also necessary to investigate larger and more heterogeneous samples (in healthy subjects as well as clinical populations), to assess long-term efficacy measures (eg, including follow-ups of 1 or more years), and to better establish specific effects of this particular well-being mobile-based program.

Moreover, it would be interesting to test this protocol against the gold standard in the field (ie, face-to-face interventions) and assess which training format works best for which type of participant and under which circumstances. Although both formats may be equally effective, they may work differently on participants with different personal characteristics, and mobile-based interventions may be more advantageous in terms of efficiency and costs.

It is important to note that no single organization regulates parameters of app efficacy or transparency. The abundance of apps that have promising descriptions but are ultimately not well developed or maintained contributes to app overload and renders people vulnerable to misdirection and discouragement as they attempt to select and adopt effective self-management strategies. Well-planned studies will improve our ability to reach the potential of mobile mental health on both individual and population levels.

### Conclusions

Our results indicate that the well-being and stress reduction app was better than the active control app at reducing employee stress and improving well-being levels. This trial contributes to the limited evidence available regarding the feasibility and efficacy of mobile-supported stress management and well-being interventions. Moreover, it is the first study to include an active control app group. The well-being mobile app presented here was highly effective in reducing perceived stress and improving mental well-being indices over a period of 4 and 8 weeks among women working at a private hospital. Thus, self-care mobile-based interventions may be used as preventive, easily accessible, and nonstigmatizing tools in a public health environment.

## Appendix

Multimedia Appendix 1 CONSORT-EHEALTH checklist (V 1.6.1).

Multimedia Appendix 2 Screenshots of the well-being mobile app, showing the app on Apple’s App Store, user log-in page, pop-up messages, evaluations, home page, brief theoretical page, guided practice timer, and profile page. Image was produced by the first author.

Multimedia Appendix 3 Screenshots of the active control app, showing the app on Apple’s App Store, user log-in page, pop-up messages, evaluations, home page, and pre- and post evaluation separated by a 20-min interval. Image was produced by first author.

Multimedia Appendix 4 Baseline outcome score data by experimental group.

Multimedia Appendix 5 Preintervention × midintervention between-group comparisons.

Multimedia Appendix 6 Preintervention × midintervention × postintervention between-group comparisons.

## Abbreviations

∆SL: stress level variation
∆WBL: well-being level variation
ANOVA: analysis of variance
AWS: Amazon Web Services
CI: confidence interval
CONSORT: Consolidated Standards of Reporting Trials
eHealth: electronic health
EMI: ecological momentary intervention
MBIs: mindfulness-based interventions
MHapps: mental health apps
mHealth: mobile health
PSS: perceived stress scale
PSS-10: 10-item perceived stress scale
WHO: World Health Organization
WHO-5: 5-item World Health Organization Well-Being Index
WMW: Wilcoxon-Mann-Whitney test
