# Supplementary figures and images for: A New Mental Health Mobile App for Well-Being and Stress Reduction in Working Women: Randomized Controlled Trial
Source: J Med Internet Res. 2019 Nov 7;21(11):e14269. doi: 10.2196/14269 (PMC6873146; doi:10.2196/14269)

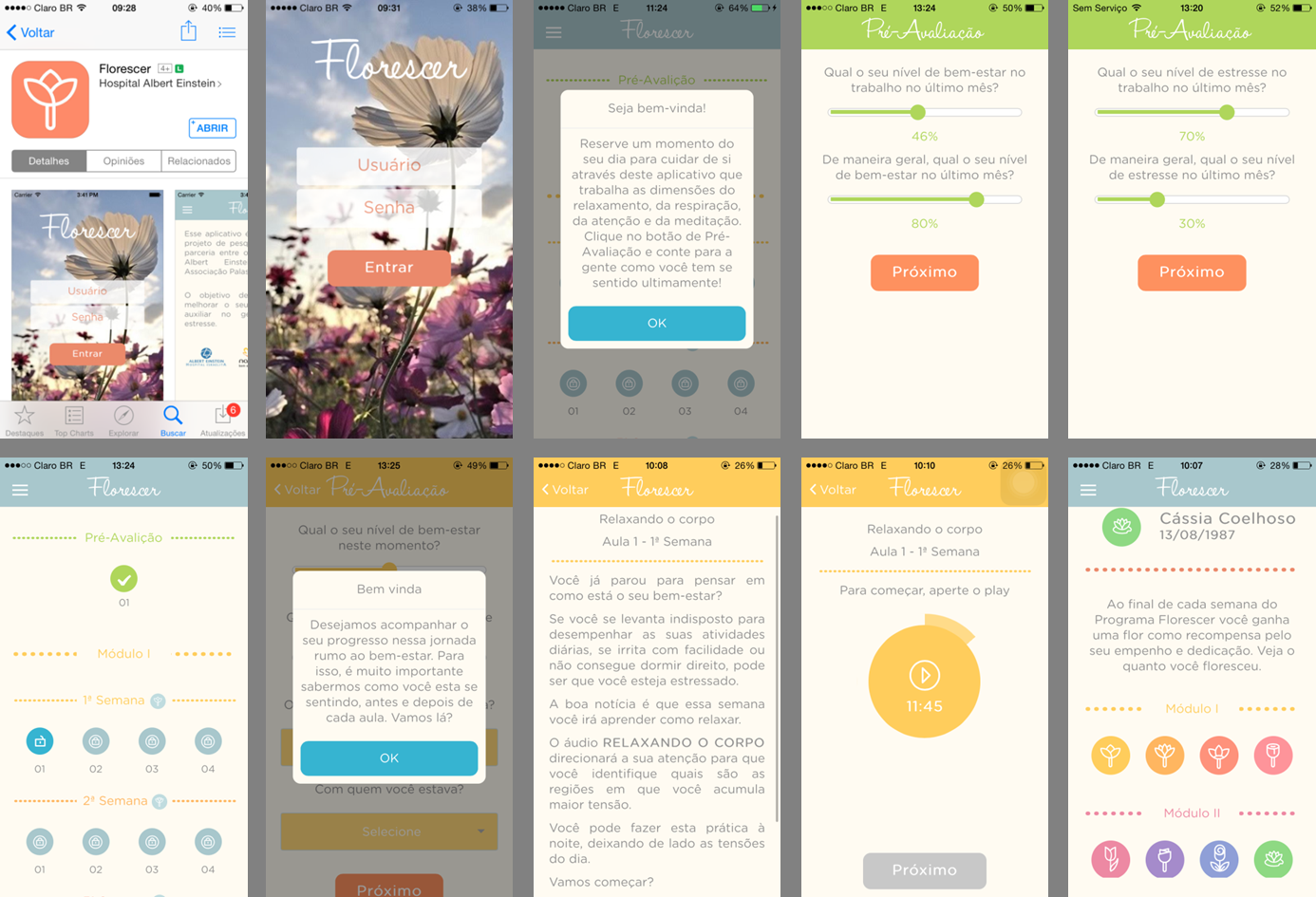

Supplement: Multimedia Appendix 2 [file jmir_v21i11e14269_app2.png]

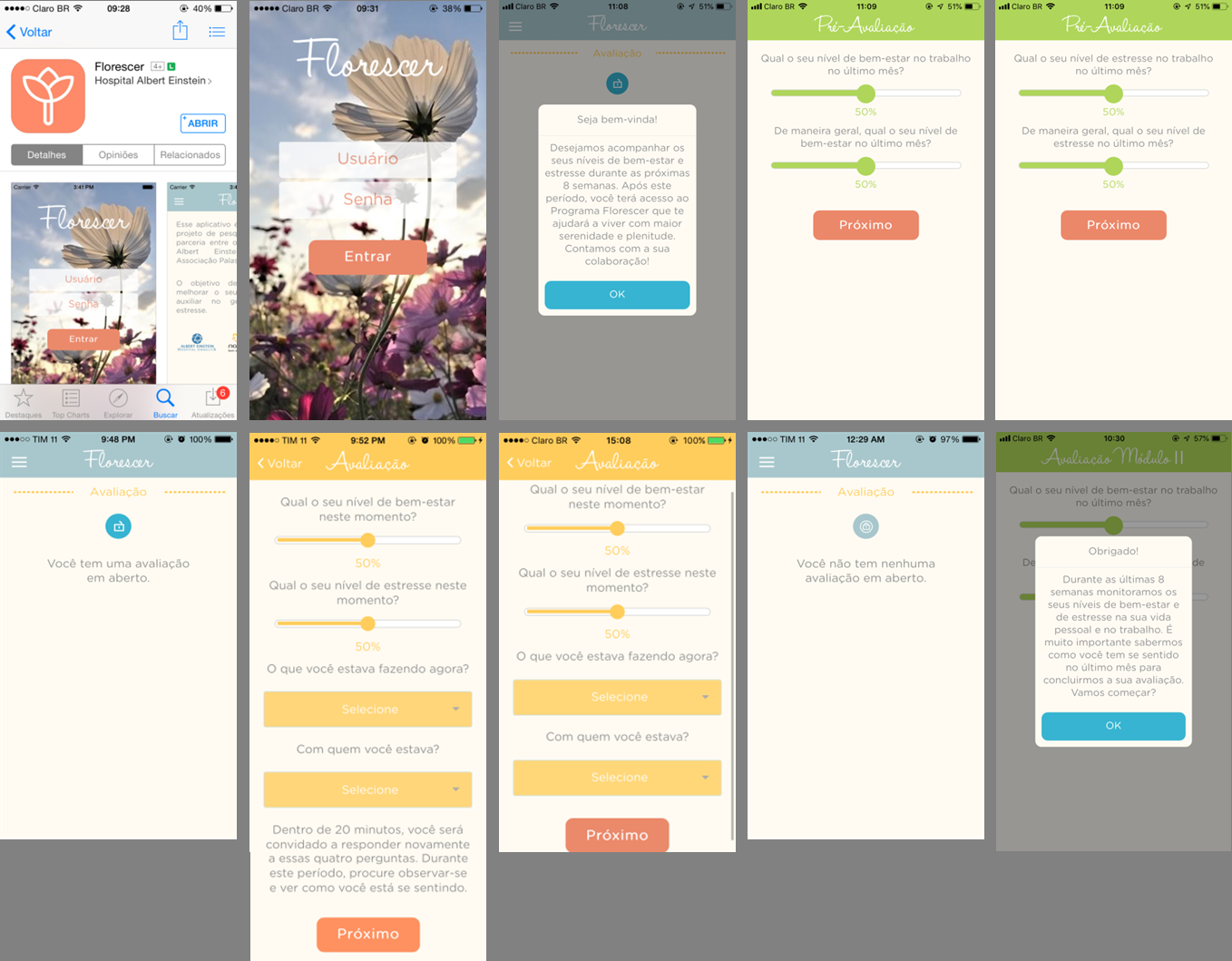

Supplement: Multimedia Appendix 3 [file jmir_v21i11e14269_app3.png]
