# Supplementary material for: A New Mental Health Mobile App for Well-Being and Stress Reduction in Working Women: Randomized Controlled Trial
Source: J Med Internet Res. 2019 Nov 7;21(11):e14269. doi: 10.2196/14269 (PMC6873146; doi:10.2196/14269)
Supplement: Multimedia Appendix 4 [file jmir_v21i11e14269_app4.pdf]

Multimedia Appendix 4

Multimedia Appendix 4. Baseline outcome score data by experimental group

	N	Control	N	Intervention	Significance
Work-related stress	157	50 (50-73.5)	148	50 (50-78.7)	*U* = 11078, *P* = .471, *r* = -0.041
General stress	157	50 (50-70.5)	148	50 (50-79.5)	*U* = 11274, *P* = .645, *r* = -0.026
Work-related well-being	157	50 (38-70)	148	50 (43.5-70)	*U* = 11487, *P* = .861, *r* = -0.010
General well-being	157	50 (48.5-68)	148	50 (45.2-66.5)	*U* = 11060, *P* = .452, *r* = -0.043
PSS-10	151	22.9 (6.24)	139	22.6 (7.16)	*t*(288) = 0.414, *P* = .679, *d* = 0.045
WHO-5	151	10 (7-14)	139	10 (7-15)	*U* = 10461, *P* = .963, *r* = -0.002

PSS-10 Perceived Stress Scale, *WHO-5* World Health Organization Well-Being Index. Data are presented as medians (25th – 75th percentile) for all outcome measures, except for PSS-10, which is presented as means (standard deviations). *U* = Mann-Whitney U test. *r* = effect size correlation for Mann-Whitney U test. *t* = Student's t-test. *d* = Cohen's *d* effect size for t-test.
