# Supplementary material for: A New Mental Health Mobile App for Well-Being and Stress Reduction in Working Women: Randomized Controlled Trial
Source: J Med Internet Res. 2019 Nov 7;21(11):e14269. doi: 10.2196/14269 (PMC6873146; doi:10.2196/14269)
Supplement: Multimedia Appendix 5 [file jmir_v21i11e14269_app5.pdf]

Multimedia Appendix 5

Multimedia Appendix 5. Preintervention x midintervention between-group comparisons

	Control		Intervention		Group difference	Time Effect		Group Effect		Time * Group Effect	
	N	Mean (SEM)	N	Mean (SEM)	Mean (95% CI)[a]	Sig.	η_p^2	Sig.	η_p^2	Sig.	η_p^2
**PSS-10**											
Pre	126	22.73 (0.61)	84	21.96 (0.75)	2.048[b]	*F* = 41.9	.168	*F* = 5.71	.027	F = 9.48	.044
Mid		21.32 (0.59)		17.99 (0.73)	(0.359 - 3.74)	*P* < .001		*P* = .018		*P* = .002	
**WHO-5**											
Pre	126	10.65 (0.46)	84	10.76 (0.56)	-1.075	*F* = 35.4	.146	*F* = 3.14	.015	F = 8.54	.039
Mid		11.65 (0.42)		13.69 (0.51)	(-2.27 - 0.122)	*P* < .001		*P* = .078		*P* = .004	
**Work-related stress**											
Pre	151	56.56 (1.73)	131	59.42 (1.86)	1.506	*F* = 2.54	.009	*F* = .452	.002	F = 6.34	.022
Mid		58.16 (2.11)		52.28 (2.27)	(.2.90 - 5.92)	*P* =.112		*P* = .502		*P* = .012	
**General stress**											
Pre	151	56.35 (1.72)	131	59.07 (1.84)	1.498	*F* = 10.4	.036	*F* = .528	.002	F = 6.46	.023
Mid		55.23 (1.89)		49.51 (2.03)	(-2.56 - 5.56)	*P* = .001		*P* = .468		*P* = .012	
**Work-related well-being**											
Pre	151	52.64 (1.66)	131	52.50 (1.79)	-5.326[b]	*F* = 67.2	.194	*F* = 6.87	.024	F = 12.1	.041
Mid		60.04 (1.83)		70.82 (1.97)	(-9.33 - -1.32)	*P* < .001		*P* = .009		*P* = .001	
**General well-being**											
Pre	151	53.85 (1.53)	131	53.20 (1.64)	2.688	*F* = 18.5	.062	*F* = 1.67	.006	F = 1.48	.005
Mid		63.08 (2.07)		58.36 (2.22)	(-1.41 – 6.79)	*P* < .001		*P* = .198		*P* = .225	

[a]Mean Difference (Control - Intervention); adjustment for multiple comparisons (Bonferroni). [b]The mean difference is significant at the .05 level. *PSS-10* Perceived Stress Scale, *WHO-5* World Health Organization Well-Being Index. Data are presented as means (standard error of mean) for all outcome measures. CI, confidence interval. η_p^2, effect size partial eta-squared.
