# Supplementary material for: A New Mental Health Mobile App for Well-Being and Stress Reduction in Working Women: Randomized Controlled Trial
Source: J Med Internet Res. 2019 Nov 7;21(11):e14269. doi: 10.2196/14269 (PMC6873146; doi:10.2196/14269)
Supplement: Multimedia Appendix 6 [file jmir_v21i11e14269_app6.pdf]

Multimedia Appendix 6

Multimedia Appendix 6. Preintervention x midintervention x postintervention between-group comparisons

	Control		Intervention		Group difference	Time Effect		Group Effect		Time * Group Effect	
	N	Mean (SEM)	N	Mean (SEM)	Mean (95% CI) [a]	Sig.	η_p^2	Sig.	η_p^2	Sig.	η_p^2
**PSS-10**											
Pre		22.78 (0.71)		21.75 (0.85)							
Mid	97	21.14 (0.69)	68	18.06 (0.82)	2.890* (1.03 – 4.75)	*F* = 45.0 *P* < . 001	.216	*F* = 9.38 *P* = .003	.054	F = 7.19 *P* = .001	.042
Post		20.15 (0.69)		15.60 (0.83)							
**WHO-5**											
Pre		10.68 (0.53)		10.69 (0.64)							
Mid	97	11.70 (0.48)	68	13.74 (0.57)	-1.609* (-2.89 - -0.33)	*F* = 33.4 *P* < . 001	.170	*F* = 6.13 *P* = .014	.036	F = 7.97 *P* = . 001	.047
Post		12.14 (0.47)		14.93 (0.56)							
**Work-related stress**											
Pre		55.88 (2.07)		59.58 (2.12)							
Mid	110	58.18 (2.53)	105	51.94 (2.59)	4.297 (-0.36 – 8.95)	*F* = 1.09 *P* =.337	.005	*F* = 3.31 *P* = .070	.015	F = 5.50 *P* = .004	.025
Post		60.02 (2.59)		49.97 (2.65)							
**General stress**											
Pre		56.20 (2.09)		59.07 (2.14)							
Mid	110	55.77 (2.25)	105	49.68 (2.30)	5.635* (1.18 – 10.1)	*F* = 15.3 *P* < . 001	.067	*F* = 6.22 *P* = .013	.028	F = 8.59 *P* < . 001	.039
Post		53.44 (2.42)		39.76 (2.48)							
**Work-related well-being**											
Pre		54.93 (1.90)		54.18 (1.95)							
Mid	110	61.29 (2.11)	105	72.77 (2.15)	-7.135* (-11.5 - -2.78)	*F* = 58.5 *P* < . 001	.215	*F* = 10.4 *P* = .001	.047	F = 8.92 *P* < . 001	.040
Post		66.09 (2.05)		76.76 (2.10)							
**General well-being**											
Pre		56.43 (1.78)		53.73 (1.82)							
Mid	110	63.49 (2.45)	105	59.41 (2.51)	4.854* (0.33 – 9.37)	*F* = 5.27 *P* = .006	.024	*F* = 4.48 *P* = .035	.021	F = 0.74 *P* = .471	.003
Post		64.71 (2.77)		56.92 (2.84)							

[a]Mean Difference (Control - Intervention); adjustment for multiple comparisons (Bonferroni). *The mean difference is significant at the .05 level. *PSS-10* Perceived Stress Scale, *WHO-5* World Health Organization Well-Being Index. Data are presented as means (standard error of mean) for all outcome measures. CI, confidence interval. η_p^2, effect size partial eta-squared.
